# Glomerular hyperfiltration and hypertrophy: an evaluation of maximum values in pathological indicators to discriminate “diseased” from “normal”

**DOI:** 10.3389/fmed.2023.1179834

**Published:** 2023-07-13

**Authors:** Hiroshi Kataoka, Kosaku Nitta, Junichi Hoshino

**Affiliations:** Department of Nephrology, Tokyo Women’s Medical University, Tokyo, Japan

**Keywords:** chronic kidney disease, glomerular hyperfiltration, glomerular hypertrophy, obesity, sodium-glucose cotransporter 2 inhibitors, visceral fat, inflammation, extreme value

## Abstract

The success of sodium-glucose cotransporter 2 inhibitors and bariatric surgery in patients with chronic kidney disease has highlighted the importance of glomerular hyperfiltration and hypertrophy in the progression of kidney disease. Sustained glomerular hyperfiltration and hypertrophy can lead to glomerular injury and progressive kidney damage. This article explores the relationship between obesity and chronic kidney disease, focusing on the roles of glomerular hyperfiltration and hypertrophy as hallmarks of obesity-related kidney disease. The pathological mechanisms underlying this association include adipose tissue inflammation, dyslipidemia, insulin resistance, chronic systemic inflammation, oxidative stress, and overactivation of the sympathetic nervous system, as well as the renin-angiotensin aldosterone system. This article explains how glomerular hyperfiltration results from increased renal blood flow and intraglomerular hypertension, inducing mechanical stress on the filtration barrier and post-filtration structures. Injured glomeruli increase in size before sclerosing and collapsing. Therefore, using extreme values, such as the maximal glomerular diameter, could improve the understanding of the data distribution and allow for better kidney failure predictions. This review provides important insights into the mechanisms underlying glomerular hyperfiltration and hypertrophy and highlights the need for further research using glomerular size, including maximum glomerular profile, calculated using needle biopsy specimens.

## 1. Introduction

Numerous studies have shown that obesity is a significant risk factor for chronic kidney disease (CKD) (1–4) and end-stage kidney disease (5–8). The link between obesity and CKD has been extensively studied, with evidence showing that excess weight and body fat can significantly damage the kidneys over time (9–11). The global spread of obesity has become a pandemic that cannot be ignored in the context of CKD (12–14), as obesity aggravates the prognosis of all kidney diseases, regardless of etiology (13, 15). Furthermore, all patients with CKD have a latent obesity risk due to lifestyle disturbances; as such, CKD and obesity are becoming inseparable. Importantly, the reno-protective effects of sodium-glucose cotransporter 2 (SGLT2) inhibitors (16–20) and bariatric surgery (9, 21–23) have been recently confirmed. Nephrologists are very interested in SGLT2 inhibitors, as, by improving glomerular hyperfiltration, these drugs significantly improve kidney disease prognosis (19, 20). At present, the significance of glomerular hyperfiltration (21, 24–26) and its pathological glomerular hypertrophy (21, 22, 27, 28) within kidney disease have returned to the spotlight. This mini-review focuses on glomerular hyperfiltration and hypertrophy, which are hallmarks of obesity-related kidney diseases; it also discusses the utility of the maximal glomerular diameter (MaxGD) as their clinical indicator and the purpose of glomerulometry in kidney pathology.

## 2. Visceral fat accumulation and CKD

Increased visceral fat accumulation can result in adipose tissue inflammation and adipokine dysregulation (29–33), leading to dyslipidemia, chronic systemic inflammation (31, 34, 35), oxidative stress (29), insulin resistance (31, 36), stimulation of the brain melanocortin system (34, 37), overactivation of the sympathetic nervous system (37–39), overactivation of the renin-angiotensin aldosterone system (40–44), mineralocorticoid receptor activation (45), sodium retention (46, 47), and expansion of the extracellular fluid volume (47–49). These conditions have complex interactions, ultimately leading to kidney damage by causing glomerular hyperfiltration (50, 51) and inflammation (52, 53), which are characteristics of obesity-related kidney disease (15, 47, 54–58).

## 3. Glomerular hyperfiltration and increased tubular sodium reabsorption in obesity

Glomerular hyperfiltration, one of the predominant pathophysiological features in obesity-related glomerulopathy (ORG) (50, 51, 59–64), is histologically characterized by glomerular hypertrophy with tubular hypertrophy (51, 65). Generally, glomerular hyperfiltration is a result of the early changes in intrarenal hemodynamic function, including increased renal blood flow and intraglomerular hypertension (66), in response to various stimuli, such as a high-protein intake (67, 68), hyperglycemia or insulin resistance (69, 70), and obesity or metabolic syndrome (50, 71–73). In obesity, renal plasma flow increases less than glomerular filtration rate (GFR) (50, 62, 71), suggesting renal vasodilation mostly affects the glomerular afferent arteriole (51). Specifically, glomerular hyperfiltration leads to mechanical stress at the filtration barrier (50, 51, 62, 65, 71, 74), stretching the glomerular capillary wall and resulting in various conditions, such as podocyte loss, mesangial expansion and sclerosis, and glomerular hypertrophy. In turn, these will increase the Bowman’s space volume, that is, a large renal corpuscle (65, 74, 75). Additionally, increased proximal tubular flow due to glomerular hyperfiltration increases delivery and reabsorption of water and solutes, causing proximal tubular epithelial hypertrophy (65), proximal tubular lumen enlargement (65), and tubulointerstitial inflammation and fibrosis (50, 51). The increased proximal reabsorption of solutes results in a decreased solute delivery to the macula densa, which in turn influences tubuloglomerular feedback, inducing preglomerular vasodilation and glomerular hyperfiltration similarly to a ‘vicious cycle’ (57, 61). These changes result in the initiation and progression of kidney disease. An elevated single-nephron GFR is associated with obesity, as well as larger nephrons, glomerulosclerosis, and arteriosclerosis on kidney biopsy and risk factors for CKD (76).

## 4. Glomerular hypertrophy and histopathological features of ORG

In patients with obesity or metabolic syndrome, relevant renal histopathological characteristics include glomerular hypertrophy with an enlargement of Bowman’s space, mesangial cell or matrix proliferation, podocytopathy, glomerular basement membrane thickening, focal segmental glomerulosclerosis (FSGS), global sclerosis, tubular hypertrophy, tubular atrophy, interstitial fibrosis, arterial sclerosis, arterial hyalinosis, and focal dilatation of the afferent arteriole and glomerular perihilar capillaries (50, 57, 63, 77, 78). Furthermore, lipid accumulation in the glomeruli and tubule cells has been confirmed in patients with obesity (3, 57, 79–81). Among these histological parameters, the current gold standard histologic features of ORG in humans are glomerular hypertrophy, large renal corpuscles, podocyte stressor FSGS, and tubular hypertrophy, which imply glomerular hypertension and hyperfiltration (50, 57, 80, 82, 83). Glomerular hypertrophy or a large renal corpuscle has been reported as a potent kidney prognostic indicator in clinical practice (84–87) and plays an important role in patients with ORG as a simple and helpful indicator that incorporates early-to-late disease stages (57, 75, 80, 87). Conversely, while FSGS may indicate the disease severity or long-term kidney prognosis, it is a somewhat nonspecific indicator of kidney disease in relation to showing the result of kidney damage (86). The challenge in tubular hypertrophy is determining which renal tubules should be measured among the myriad of renal tubules in renal biopsy tissue. Furthermore, tubular hypertrophy assessment is complicated by the fact that increased severity of coexisting interstitial fibrosis and tubular atrophy in biopsy specimens are associated with a worse kidney outcome (88–90). Primarily, glomerular hypertrophy or a large renal corpuscle (52, 75, 84–87, 91) represents both glomerular hyperfiltration and glomerular inflammation and is recognized as an early marker of obesity-related kidney damage (52, 63). Considering the prophylactic significance of early indicators in patients with obesity, increased clinical use of indicators of glomerular hypertrophy or a large renal corpuscle is desired.

## 5. Discussion

### 5.1. Pathological assessment of glomerular hyperfiltration

Kidney biopsy can help identifying the underlying cause of kidney disease and assess disease progression and treatment effectiveness (92–94). Various histological indices can be used to evaluate glomerular hyperfiltration (65, 75, 84–86, 90, 95–101). Especially, glomerular hypertrophy assessment is thought to be the most clinically significant (84–86, 90, 99, 100). First, glomerular hypertrophy is a direct response to glomerular hyperfiltration and represents the earliest histological change (75, 102). As reported, glomerular hypertrophy and renal hypertrophy are observed soon after the onset of type 1 diabetic nephropathy (103), and persistent renal hypertrophy precedes the development of microalbuminuria and GFR decrease (102). Second, although albuminuria and proteinuria are the leading prognostic factors for CKD and progression to end-stage kidney disease (104, 105), glomerular hypertrophy is associated with albuminuria/proteinuria and kidney function decline to a greater extent than other parameters (80, 84, 86), making it a clinically important parameter to evaluate in patients with glomerular hyperfiltration. Therefore, glomerular hypertrophy assessment can aid in the early detection and monitoring of kidney disease, as well as in the development of targeted treatment strategies (75). Furthermore, glomerular hypertrophy evaluated by glomerular diameter or glomerular area measurements is less prone to measurement variability, and can easily provide a more precise evaluation of glomerular hyperfiltration than tubular measurements, which are more prone to variation in tubular diameter and length (75, 89).

### 5.2. Factors to keep in mind when evaluating glomerular hypertrophy

The size of the renal corpuscle, or glomerulus, is an important indicator of renal function and has been associated with the outcomes of kidney diseases in both experimental models (102, 103, 106, 107) and humans (84–86, 108–110). However, the currently used clinical practice guidelines and pathological classifications of glomerulonephritis do not adequately evaluate glomerular or renal corpuscle sizes as markers of a renal lesion (75, 83, 111). When using glomerular hypertrophy as an effective clinical indicator, clinicians should distinguish between physiological and pathological glomerular hypertrophy and ensure the accuracy and consistency of measurements (27, 75). Evaluating the glomerular size in clinical studies can be challenging (84, 112) due to glomeruli heterogeneity in a single kidney (113), presence of sclerosed and collapsing glomeruli, and use of different measurement techniques. Furthermore, the use of differing morphometric techniques (114), sample sizes, and statistical processing in the evaluation of the glomerular size in previous studies may have influenced the inconsistent results in clinical research.

### 5.3. The definitions of “normal” and “diseased”

The definitions of “normal” and “diseased” play an important role in medical practice. A clinically useful diagnostic definition of “normal” is based on a measurement rage within which the disease is absent in diagnostic tests (115). In kidney disease, differentiating between morbid and physiological enlargement of renal corpuscles is important, as morbid glomerular hypertrophy may involve irreversible structural changes that can lead to renal function decline (27, 75). Physiological hypertrophy may occur as a normal response to aging or an increased demand, such as in during pregnancy. Conversely, pathological hypertrophy is a maladaptive response to various insults, such as hypertension, diabetes, and glomerulonephritis, and can result in irreversible structural changes in the kidney (27, 75). In a previous study of IgA nephropathy, glomerular hypertrophy above a maximal diameter of 242 μm was associated with follow-up proteinuria aggravation and an increase in serum creatinine levels. Enlargement of the renal corpuscle to more than 1.5 times its original diameter may be considered morbid, and this threshold effect in the renal corpuscle size may be an important consideration when evaluating renal function and disease progression (84).

### 5.4. Points to note when measuring the glomerular size or renal corpuscle size

Importantly, the use of differing morphometric techniques (114), sample sizes, and statistical analyses in the evaluation of the glomerular size in previous studies may have influenced inconsistent results in clinical research. There are two major methods used to investigate the glomerular size or renal corpuscle size in human kidneys: estimating the mean glomerular size and measuring the individual glomerular size (75, 114). The traditional model-based method of Weibel and Gomez is widely applied to estimate the mean glomerular volume in biopsy specimens (116), but it cannot quantify the heterogeneity of the glomerular volume distribution. An individual glomerular volume allows the assessment of glomerular size variability within the kidneys, and two representative methods of measuring individual glomerular volumes are the Cavalieri (117) and maximal profile area (MPA) (118) methods. The MPA method is more feasible and advisable in clinical settings, as it allows the examination of more glomeruli in small tissue samples (119), while being less laborious (114) than the Cavalieri method.

### 5.5. Mean or maximum value?

**Figure 1:**
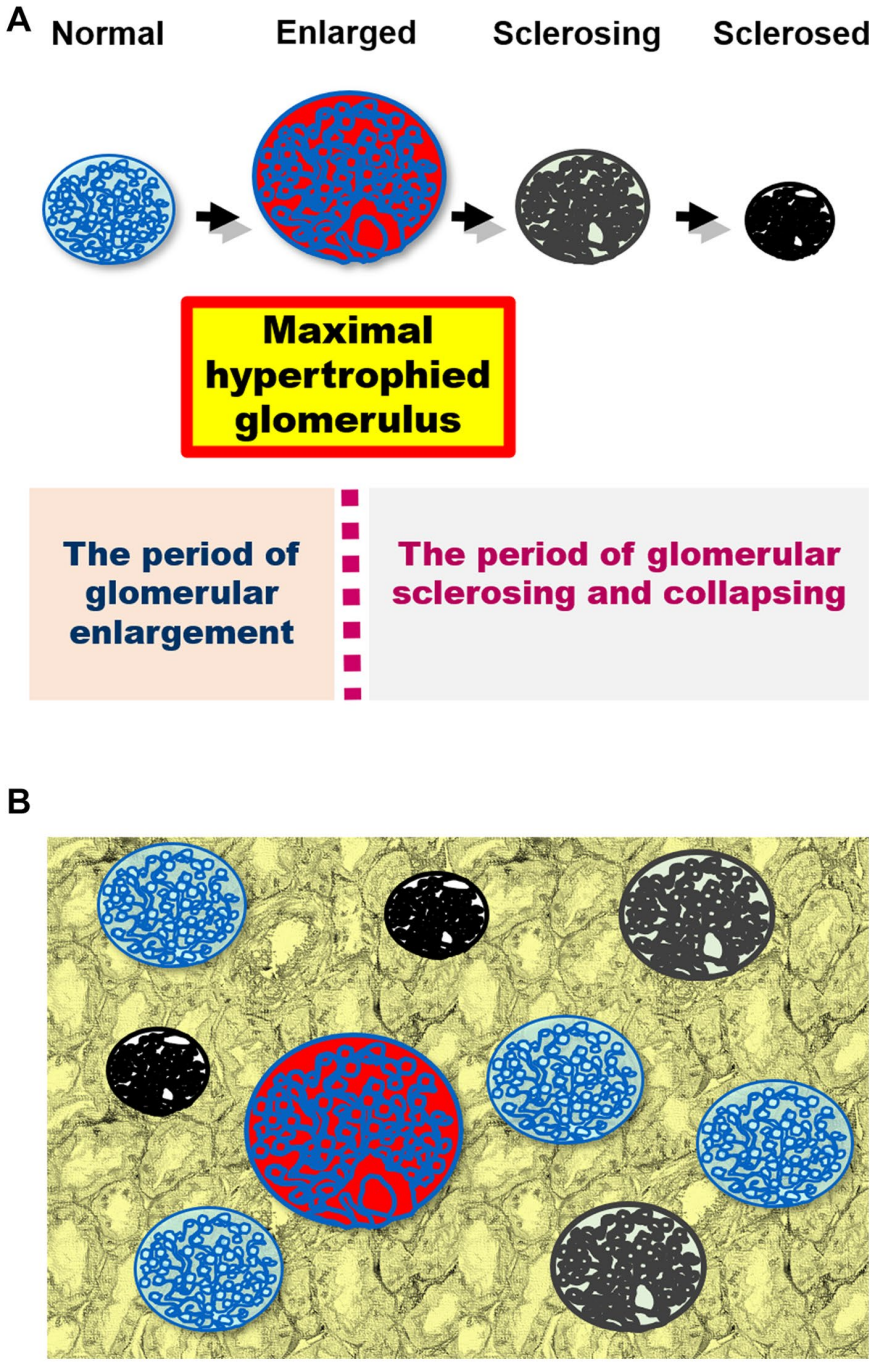
**(A)** A schematic representation of the changes in glomerular size in kidney damage. Injured glomeruli increase in size before sclerosing and collapsing. The maximal hypertrophied glomerulus is shown in red. The course can be divided into two periods using the maximally hypertrophied glomerulus as a boundary: glomerular enlargement and glomerular sclerosing and collapsing. **(B)** Glomeruli of various sizes (including hypertrophied and collapsing glomeruli) simultaneously exist in the kidney biopsy specimen.

**Figure 2:**
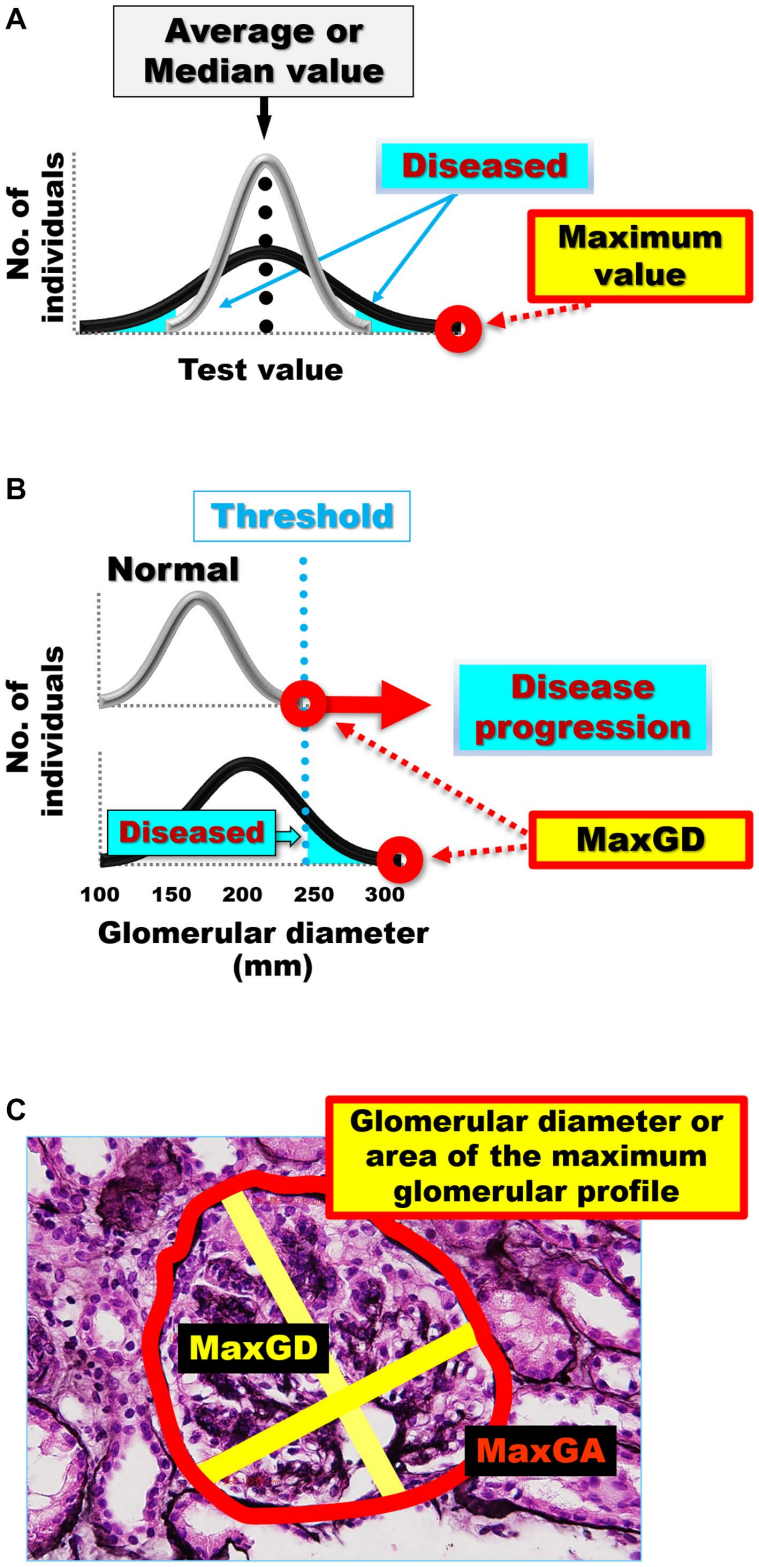
**(A)** Average or median values often cannot distinguish the differences in distribution curves for normal and diseased in diagnostic tests. The gray distribution curve represents the distribution of healthy participants in a diagnostic test, while the black distribution curve represents the distribution of patients in a diagnostic test. Differences between the black and gray distribution curves can be identified by extreme values, such as maximum and minimum values, rather than by the mean and median values. **(B)** The differences in the distribution curves of glomerular size between healthy individuals and patients with kidney damage. The gray distribution curve represents the glomerular size distribution in healthy participants without kidney damage. The black distribution curve represents glomerular size distribution in patients with kidney damage. Pathological glomeruli can be diagnosed when hypertrophy progresses beyond a certain threshold. The greater the damage to the kidney, the more the maximum glomerular profile shifts to the right in the kidney biopsy specimens. **(C)** Glomerular diameter or area of the maximum glomerular profile. Measurement of the MaxGD is indicated by the yellow line and MaxGA is represented by the red line. The MaxGD is calculated as the mean of the maximal diameter of the glomerulus and the maximal chord perpendicular to the maximal diameter of the maximally hypertrophied glomerulus in the area with the maximal profile in each specimen. The position of the geometric center of the maximal profile of the glomerulus is visually identifiable. After drawing the maximal diameter that passes through the geometric center, we draw the maximal chord perpendicular to the maximal diameter. MaxGD, maximal glomerular diameter; MaxGA, maximum glomerular area.

When measuring glomerular size (75, 84), it is important to consider that the size of each glomerulus varies over time (Figure 1A) and that glomeruli of various sizes, including hypertrophied and collapsing glomeruli, can be found within the same kidney (Figure 1B). Indeed, injured glomeruli increase in size before sclerosing and collapsing (120), and sclerosing and normal renal corpuscles with sized in the range of 160–180 μm coexisted in a study of IgA nephropathy (84). Of note, the mean glomerular size may not accurately reflect the true distribution of glomerular sizes in a kidney and may conceal important prognostic information (84). The use of extreme values (i.e., minimum and maximum values) in medical research is relatively rare, despite their applications in other fields, such as hydrology, earth sciences, environmental science, finance, insurance, and genetics, for forecasting unusual events (121). However, we consider that the basal concept of the extreme value theory can be used to predict the occurrence of events, such as kidney failure, from limited samples, such as kidney biopsy specimens (84, 121). This is because the mean and standard deviation, which are commonly used in medical research, are only appropriate for normally distributed data, which is often not the case in biological data (122, 123). Primarily, the mean and standard deviation, which are two of the most common descriptive statistic measures for continuous data and can be calculated from as few as two data points, correctly signify only a “normal” or “Gaussian” value distribution and cannot accurately describe small samples (122). The use of other statistical measures, such as the median and range or maximum values, could provide more informative insights into the distribution of data in diagnostic tests and allow for better prediction of unusual events (102, 115, 124–126) (Figure 2A). The most important phenomenon in kidney disease progression is the fact that injured glomeruli increase in size, represented by a rightward shift in the size distribution, before sclerosing and collapsing (102, 120) (Figure 2B). We consider that the difference in magnitude of a rightward shift in the glomerular size distribution caused by nephron loss, as well as by direct glomerular damage, can help distinguish between compensatory and pathological glomerular hypertrophy (75, 84). The maximum glomerular profile could be a direct indicator of the disease severity or progression, while being less susceptible to nephron loss (27, 121).

### 5.6. Maximal glomerular profile in needle biopsy specimens

We have proposed a new method that focuses on extreme values, specifically the maximal glomerular profile, such as glomerular diameter (75, 84) or area (87), to predict the progression of CKD (Figure 2C). The clinical value of our pathological evaluation method, which measures only the largest glomerulus instead of the average value of all glomeruli, has been confirmed in subsequent studies. We have demonstrated poor renal prognosis in patients with IgA nephropathy only by using the maximal renal corpuscle diameter, instead of the mean renal corpuscle diameter (84). Glomerular hypertrophy, as indicated by a MaxGD ≥242.3 μm, has been shown to be a significant predictor of poor renal prognosis in patients with IgA nephropathy (84). We also confirmed the MaxGD as an effective pathological factor for predicting renal IgA nephropathy prognosis in another cohort (86) and in a follow-up study (85). In addition, using a combination of traditional statistical methods and machine learning to identify pathophysiological factors associated with glomerular hypertrophy we demonstrated that the MaxGD is not an indicator of compensatory glomerular hypertrophy but of renal damage itself (27).

## 6. Perspective

While glomerular hyperfiltration has received more attention, there is still a lack of clinical research on its pathological index, glomerular hypertrophy. Historically, medical research assessing extreme values is not common, however, evaluating these values may help distinguish between “normal” and “diseased” states. Measurement of the MaxGD is the most clinically superior method for assessing glomerular hypertrophy in the context of glomerular hyperfiltration. This is because it provides direct information on the size of glomeruli, which can increase in response to hyperfiltration. In addition, this method is more objective, reproducible, and less influenced by interobserver variability than other methods. Overall, maximal glomerular diameter measurements offer valuable information for the pathological assessment of glomerular hyperfiltration, and can aid in the diagnosis and management of kidney diseases. Research using the maximum glomerular profile, including the MaxGD, is expected to contribute to the field of kidney disease in the future.

## Author contributions

HK performed the literature search and wrote the manuscript. KN and JH were involved in planning and supervising the work. All authors contributed to the article and approved the submitted version.

## Funding

This study was partly supported by a Grant-in-Aid for Intractable Renal Diseases Research and Research on Rare and Intractable Diseases, as well as by Health and Labor Sciences Research Grants from the Ministry of Health, Labor and Welfare of Japan.

## Conflict of interest

The authors declare that the research was conducted in the absence of any commercial or financial relationships that could be construed as a potential conflict of interest.

## Publisher’s note

All claims expressed in this article are solely those of the authors and do not necessarily represent those of their affiliated organizations, or those of the publisher, the editors and the reviewers. Any product that may be evaluated in this article, or claim that may be made by its manufacturer, is not guaranteed or endorsed by the publisher.
